# Deep neural network based quantum simulations and quasichemical theory for accurate modeling of molten salt thermodynamics

**DOI:** 10.1039/d2sc02227c

**Published:** 2022-06-15

**Authors:** Yu Shi, Stephen T. Lam, Thomas L. Beck

**Affiliations:** Department of Chemistry, University of Cincinnati Cincinnati OH 45221 USA; Department of Chemical Engineering, University of Massachusetts Lowell MA 01854 USA; National Center for Computational Sciences, Oak Ridge National Laboratory Oak Ridge TN 37830 USA becktl@ornl.gov

## Abstract

With dual goals of efficient and accurate modeling of solvation thermodynamics in molten salt liquids, we employ *ab initio* molecular dynamics (AIMD) simulations, deep neural network interatomic potentials (NNIP), and quasichemical theory (QCT) to calculate the excess chemical potentials for the solute ions Na^+^ and Cl^−^ in the molten NaCl liquid. NNIP-based molecular dynamics simulations accelerate the calculations by 3 orders of magnitude and reduce the uncertainty to 1 kcal mol^−1^. Using the Density Functional Theory (DFT) level of theory, the predicted excess chemical potential for the solute ion pair is −178.5 ± 1.1 kcal mol^−1^. A quantum correction of 13.7 ± 1.9 kcal mol^−1^ is estimated *via* higher-level quantum chemistry calculations, leading to a final predicted ion pair excess chemical potential of −164.8 ± 2.2 kcal mol^−1^. The result is in good agreement with a value of −163.5 kcal mol^−1^ obtained from thermo-chemical tables. This study validates the application of QCT and NNIP simulations to the molten salt liquids, allowing for significant insights into the solvation thermodynamics crucial for numerous molten salt applications.

## Abstract 2

Solvation thermodynamics in molten salt is accurately and efficiently predicted by combining *ab initio* molecular dynamics (AIMD) simulations, deep neural network interatomic potentials (NNIP), and quasichemical theory (QCT).

## Graphical abstract

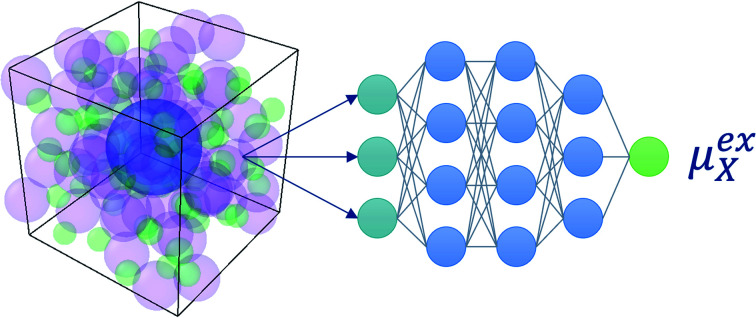

## 1 Introduction

The study of molten salt properties has seen a major resurgence in recent decades due to promising applications in clean energy technologies such as molten salt reactors^1–3^ and concentrated solar power storage.^4,5^ The diverse application of molten salts is primarily due to their usage in high-temperature heat-transfer media. Molten salts are excellent candidates in these systems due to their favorable physicochemical properties (*e.g.*, thermal conductivity, heat capacity, viscosity, *etc.*) and relatively low cost of production.^6^ When designing and optimizing salt mixture compositions for various applications, however, exploration of a high-dimensional material space is required to select for candidates with optimal properties. In addition, it is important to understand corrosion at alloy/molten-salt interfaces and property-evolution during reactor operation. This makes the experiments (such as X-ray and neutron diffraction and electrochemical measurements^7–11^) expensive and challenging under extreme conditions.

Therefore molecular dynamics (MD) simulations have been exploited to calculate molten salt properties. Classical simulations are efficient for modelling systems over timescales on the order of nanoseconds in order to make viable predictions. Empirical force-field molecular dynamics (FFMD) simulations with classical potentials such as the Born–Mayer–Huggins–Tosi–Fumi (BMHTF) rigid-ion potential^12–15^ have been demonstrated to lack full predictive capabilities due to the lack of many-body interactions that influence local structure. Polarizable ion models (PIM) developed in conjunction with quantum mechanical calculations have led to significant improvements in modeling structure, thermodynamics, and dynamic properties.^16,17^

*Ab initio* molecular dynamics (AIMD) simulations have been shown to be capable of accurately capturing local structure and solute chemistry in comparison with experiments.^18–26^ AIMD is computationally expensive, however, and therefore it is difficult to access long time and large length scales to predict the dynamic and thermal properties. Recently, state-of-the-art artificial neural network-based interatomic potential molecular dynamics simulations (NNIP-MD) have been demonstrated as a promising computational tool to explore the underlying physics of the high-dimensional molten salt compositions by simulating 10^4^ atoms on the timescale of nanoseconds with an accuracy at the level of density functional theory (DFT). It has been demonstrated that the NNIP-MD studies^27–30^ are capable of accurately predicting molten salt structure, heat capacity, self-diffusion coefficients, thermal conductivity, electrical conductivity, viscosity and the melting/freezing point in comparison with experimental measurements.

Understanding phase behavior remains a great challenge at the forefront of molten salt research. In addition, during reactor operation, there is continuous evolution due to processes such as transmutations, fission/corrosion product generation, gas bubble formation, precipitation of insoluble species and other reactions.^31,32^ A challenge facing the molten salt research community is to directly model these properties starting from phase diagrams, but many of the phase diagrams have never been constructed.

The CALPHAD method is a principal method for phase diagram and molten salt database development.^32–37^ Experimental thermodynamic data (from phase equilibria measurements and activities of solution species) and/or AIMD simulations are used to empirically fit models and then predict stable phases at different temperatures or compositions. As mentioned above it would be difficult, expensive, and time-consuming to obtain data for high-dimensional salt systems through either experiments or AIMD simulations.^19,38^

The chemical potential is central for understanding molten salt thermodynamic properties and predicting phase behavior. Studies of the chemical potential are limited due to the above-mentioned difficulties, however. The classical PIM potential force field has been used to calculate the activity coefficient ratios for lanthanide cations (*e.g.* U^3+^ to Y^3+^ in the LiCl/KCl eutectic mixture^39^) and the free energy change for the reaction of the Eu^3+^/Eu^2+^ redox couple in molten KCl.^40,41^ The Widom particle insertion method has been employed with AIMD simulations to calculate the chemical potential and the solubility of the sodium atom in molten NaCl.^42^

The cutting-edge NNIP-MD methods hold significant promise to play a vital role in exploring the thermodynamic properties since they provide quantum-level accuracy with efficiency similar to classical simulations. In the present work, molten NaCl is chosen as a prototype system to demonstrate the validity of the NNIP-based quasichemical theory (QCT) calculations, which is shown to be an efficient approach for directly calculating the solute ion solvation free energy *via* molecular dynamics simulations.^43–45^ To our knowledge, this is the first application of deep learning methods to the calculation of excess thermodynamic properties of molten salts.

## 2 Methods

### 2.1 Theoretical methods

The chemical potential of the solute X (Na^+^ or Cl^−^ ion) solvated in the molten salt liquid phase can be expressed as

1 *μ*_X_ = *μ*^id^_X_ + *μ*^ex^_X_

where *μ*^id^_X_ is the ideal contribution, and the second term *μ*^ex^_X_ is the excess chemical potential due to interactions of the solute X with the solvent ions.

The solute(X)-solvent interaction energy is defined as *ε*_X_ = *U*_solution_ − *U*_solvent_ − *U*_solute_, where *U* is the system total potential energy. Then the excess chemical potential is given by

2 *μ*^ex^_X_ = −*kT* ln〈*e*^−*ε*_X_/*kT*^〉_0_

where the angled brackets denote the ensemble average and the subscript “0” indicates that the solute X is absent during the simulation. In the QCT, the excess free energy is partitioned into three physical parts by manipulations involving insertion and withdrawal of a hard particle that carves out a cavity in the liquid:^43–45^

3 *μ*^ex^_X_ = −*kT* ln〈*e*^−*M*_*λ*_/*kT*^〉_0_+*kT* ln〈*e*^−*M*_λ_/*kT*^〉_*ε_X_*_−*kT* ln〈*e*^−*ε*_X_/*kT*^〉_*M_λ_*_

where *M*_*λ*_ is a repulsive potential (half-harmonic potential in the current work) that pushes solvent ions away to the distance *λ*. The first term (packing, PK) is the free energy change to grow a cavity of radius *λ* in the liquid. The second term (inner-shell, IS) is minus the free energy change to grow the same cavity in the liquid around the solute X and the subscript *ε*_X_ indicates that the solute X is present during the simulation. The third term (long-ranged, LR) is the free energy change for inserting the solute X into the cavity center and the subscript *M*_*λ*_ indicates that the solute X is absent and there is a cavity generated by the repulsive potential M_*λ*_ during the simulation (referred to as “uncoupled” below).

The LR contribution can also be written as^43,44^

4 *μ*^ex^_LR_ = *kT* ln〈*e*^*ε*_X_/*kT*^〉_*M*_λ_+*ε*_X__

where the subscript *M*_λ_ + *ε*_X_ indicates the solute X is present at the cavity center fully interacting with solvent ions during the simulation (referred to as “coupled” below). The PK and IS contributions are computed *via* thermodynamic integration (TI)^46^ by slowly growing in the repulsive potential M_*λ*_ using the functional form

5 *f*_*λ*_(*γ*)=*γ*^3^*M*_*λ*_,

where *γ* is the coupling parameter that evolves from 0 to 1 (in the current work the step-size is 0.1).

The repulsive potential M_*λ*_(*r*) is a half-harmonic potential6
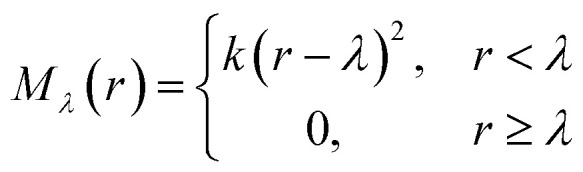
where *k* = 100 kcal mol^−1^ Å^−2^ and *λ* = 4.0 Å as exploited in previous AIMD calculations^45^ for Na^+^ ion solvation in water.

The PK contribution is then expressed as7
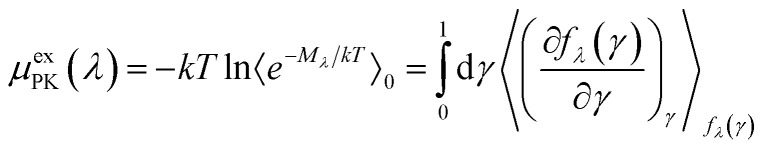
and the solute X interacts with solvent ions through the potential function *f*_*λ*_(*γ*) during the simulation, while the inner-shell contribution is given by8
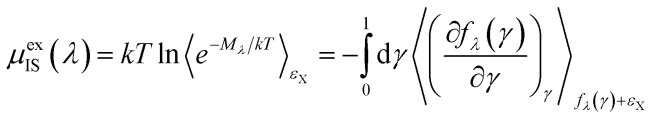
where the solute X fully interacts with solvent ions including the potential function *f*_*λ*_(*γ*) during the simulation.

For the long-ranged contribution, a cumulant expansion to second order yields the (uncoupled) expression9
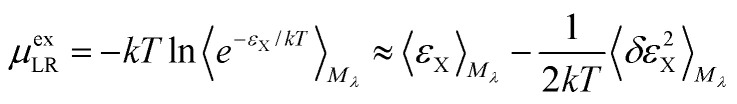
where the *δε*__X__ is the fluctuation term for the solute–solvent ions interaction energy. The corresponding coupled expression is10
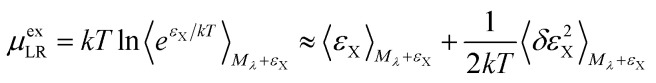


The regularization^47^ due to the repulsive potential M_*λ*_ (that pushes the solvent molecules/ions away from the solute) produces near-Gaussian statistics for *ε*_X_, and thus the two fluctuation terms of the uncoupled (eqn (9)) and the coupled (eqn (10)) samplings^44^ are of close magnitude. Consequently, the average of the two mean terms gives a relatively accurate approximation for the LR contribution.

Alternatively, at a value for which the two interaction energy distribution functions are equal,^43^11
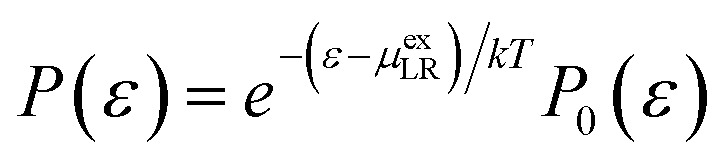
the LR free energy contribution *μ*^ex^_LR_ is exactly equal to the interaction energy *ε* at the intersection point,^46,48,49^ and we utilize this equality below. Above *P*_0_(*ε*) is the uncoupled distribution and *P*(*ε*) is the coupled distribution.

### 2.2 Computational methods

In the following section, we discuss the implementation of the AIMD simulations with CP2K 2.6.1,^50^ the training process for the NNIP model with DeePMD-Kit,^51,52^ and the protocol for the NNIP-MD simulations with LAMMPS.^53^

#### 2.2.1 AIMD simulation setup

Using the QuickStep module of the CP2K package, we performed all the DFT-based simulations of molten NaCl with 64 solvent ion pairs and 1 solute ion (Na^+^ or Cl^−^) fixed at the center of a periodic cubic box. The box size is determined by 
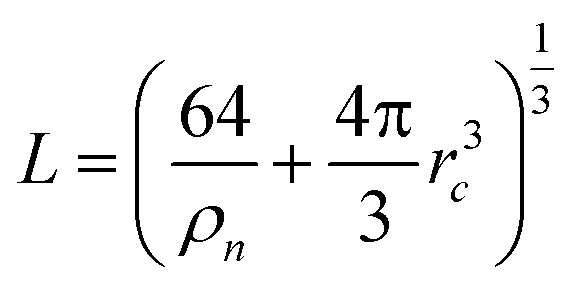
, where the ion number density is *ρ*_*n*_ = 15.61 (nm)^−3^,^42^ and *r*_*c*_ = 4.0 *γ* Å. The coupling parameter *γ* is varied from 0 to 1 during the thermodynamic integration for the PK and IS contributions.

The initial configurations were generated during classical molecular dynamics simulations using the GROMACS 4.5.5 package,^54^ and the ions were modeled with the OPLS-AA force-field.^55^ With 1 fs as the time step, all classical force-field based simulations were run for 1.5 ns in the NVT ensemble after 1 ns of equilibration in the NPT ensemble. The temperature was set at 1150 K for the classical simulations. For the AIMD simulations, parameters were chosen based on previous studies that accurately predicted local structure, standard reduction potential and sodium solubility in molten NaCl.^42^ This includes the dual basis sets of Gaussian-type orbitals (double zeta bases, DZVP-GTH) and plane waves with a 600 Ry cutoff.^56^ Atomic cores were modeled with the Goedecker-Teter-Hutter pseudopotentials (GTH).^57^ The Perdew–Burke–Ernzerhof (PBE)^58^ functionals were used for all atoms in the system, and the D2 dispersion corrections^59,60^ were utilized. D2 dispersion corrections were also employed in a recent NNIP study on molten NaCl liquid.^28^ While more advanced dispersion methods such as D3 could be used,^61^ systematic improvement in predictions across different properties is difficult to achieve, due to the semi-empirical nature of most computationally efficient DFT-based dispersion methods. In any case, from density predictions in molten NaCl,^42^ we find that additional corrections based on higher-level theory are likely necessary to achieve chemical accuracy.

The Ewald potential^45,62,63^ was applied for the electrostatic interactions under periodic boundary conditions (PBC), and a Nosé-Hoover thermostat chain^64^ of length 3 was coupled to each ion to maintain a temperature of 1150 K for all the NVT ensemble simulations. Due to the requirement of many simulations along the thermodynamic integration paths to grow the nano-scale cavities, we were not able to employ the path integral formalism for incorporating quantum effects.^65^ These corrections are expected to be small at such a high temperature, however.

The time step was taken as 0.5 fs. For the LR contribution, two 25 ps simulations (50 000 configurations, coupled and uncoupled) were implemented. This is found to be sufficient for calculating ensemble-average energies based on previous AIMD studies with molten salts.^26–28,42^ We performed 9-step integrations for the PK and IS calculations, and we ran simulations for 10 ps (20 000 configurations) for each step.

#### 2.2.2 DNN setup for potential energy surface and forces

In the DeePMD-kit framework, a local coordinate frame should be constructed to preserve translational, rotational, and permutational (same species exchange) symmetry.^51^ We set up the axes with the first axis along the direction to the nearest atom of the same ion type and the second axis along the direction to the nearest atom of the other ion type. Within this local coordinate system, the (*x*_ij_,*y*_ij_,*z*_ij_) are the Cartesian components of the distance vector *R*_ij_, where atom j is a generic neighbor of atom i. The full radial and angular information around atom i is included as *D*_*ij*_ = {1/*R*_ij_,*x*_ij_/*R*^2^_ij_,*y*_*ij*_/*R*^2^_ij_,*z*_ij_/R^2^_ij_} for the closest 20 atoms and only radial information is included as *D*_ij_ = {1/R_ij_} for up to 40 more atoms within the cutoff distance of *R*_c_ = 6.8 Å. Full details of the NNIP can be found in previous studies.^51,52^ The descriptors of each atom are used as inputs into a feed-forward DNN of 5 hidden layers with decreasing numbers of neurons, (512, 256, 128, 32, 8). Each neuron takes data D^in^_l_ from the previous layers and outputs D^out^_k_ to the next layer, implementing the linear transformation *D̃*_k_ = ∑_*l*_*w*_kl_*D*^*in*^_*l*_ + b_*k*_, followed by the non-linear transformation *D*^out^_k_ = *φ*(*D̃*_k_), where the hyperbolic tangent function *φ* is used as the activation function. In the last layer, only the linear transformation is applied to produce atomic energy *E*_i_. The sum of all atomic energies then yields the total energy *E*. The forces on each atom were computed as negative derivatives with respect to position. The loss function was taken as12
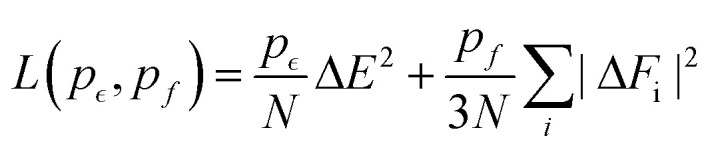
where Δ*E* and Δ*F*_i_ are the root mean square (RMS) errors of the energy of the system and the force *F* on atom i, *N* is the number of atoms, and *p*_*ε*_ and *p*_*f*_ are the adjustable pre-factors. As the training proceeded, *p*_*ε*_ began at 0.02 and ended at 8, and *p*_*f*_ changed from 1000 to 1. The initial learning rate was 0.0001 with a decay rate of 0.95 for 5000 total decay steps. The Adam stochastic gradient descent method^66^ was used to minimize the loss function and find the parameters *w*_kl_ and *b*_k_ of each hidden layers. The batch-size was 5 and the training process proceeded for 1 000 000 steps. The training data consisted of energies and forces from AIMD simulations.

From the AIMD simulations, we generated 20 000 configurations for each of the first 9 steps of the thermodynamic integration. For the last step, where *γ* = 1, 50 000 configurations were collected from the two coupled simulations (IS of Na^+^ and Cl^−^) and one uncoupled simulation (PK), respectively. We trained a model for each step of the TI process (IS and PK, 10 models in total) and another 2 models for the interaction energy calculations over the uncoupled and coupled configurations of the LR simulations, respectively. We also tried to obtain a single NNIP model by using all of the collected AIMD data. This all-in-one model produced an error of magnitude about 3 kcal mol^−1^ for the solute–solvent interaction energy compared with the AIMD calculation, and a similar error in the free energy. Due to this relatively large error, we did not utilize the all-in-one method further.

#### 2.2.3 NNIP-MD simulation setup

DeePMD-kit provides LAMMPS support through a third-party package in order to produce classical MD simulations using the NNIP to compute the atomic interactions. In this way, large time-scale simulations are accessible with quantum accuracy.^51,52^ The box size is determined in the same way as shown in the AIMD simulation setup discussion. We ran NVT simulations using the LAMMPS code for systems of each ion in the molten salt liquid. Following the calculation for the Na^+^ ion hydration free energy,^45^ a cavity of radius 4.0 Å was included at the periodic box center. The variations in each contribution due to the change of the cavity size are discussed in detail in our previous calculations.^44^ We applied a Nosé-Hoover thermostat chain of length 3 to maintain a temperature of 1150 K. The system sizes were calculated following the above method. The NNIP-MD simulations were run for 1000 ps with the first 250 ps for equilibration and the subsequent 750 ps for data production. A time step of 0.5 fs was utilized and the configurations were recorded every 0.01 ps (every 20 steps).

## 3 Results and discussion

**Fig. 1:**
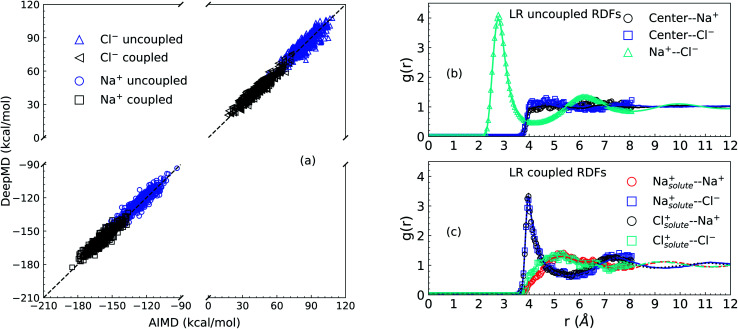
Validation of NNIP-MD simulations in comparison with AIMD simulations. Panel (a) is the NNIP-MD interaction energy extrapolation. The energies are calculated for 1000 configurations sampled with/without a (coupled/uncoupled) solute ion (Na^+^/Cl^−^) at the center of a cavity of radius 4 Å in the molten NaCl liquid of 64 solvent ion pairs. Panel (b) and (c) are radial distribution functions (*g*(*r*)) calculated over the configurations for LR contributions, where curves are from NNIP-MD simulations. Symbols are from AIMD simulations. Panel (b) is for the uncoupled sampling (without the solute ion centered in the cavity of 4 Å). Panel (c) is for the coupled sampling (with the solute ion at the center of the cavity).

We first present results related to the NNIP model validation. For the LR contributions, the solute–solvent ion interaction energies calculated *via* AIMD and NNIP-MD simulations are shown in Panel (a) of Fig. 1. Averaging over 11000 configurations drawn from NNIP-MD simulations for the systems of one solute ion and 64 solvent ion pairs, the interaction energy of the solute Na^+^ calculated *via* NNIP exhibits a 1.4 kcal mol^−1^ deviation from AIMD for the uncoupled simulation and 1.3 kcal mol^−1^ deviation for the coupled case. The deviations for the solute Cl^−^ are −0.4 kcal mol^−1^ (uncoupled) and 0.4 kcal mol^−1^ (coupled). These values are close to chemical accuracy (∼1 kcal mol^−1^) and indicate sufficient accuracy of the NNIP model for the free energy calculations.

Next, as shown in Panel (b) and Panel (c) of Fig. 1, the overlapping of radial distribution functions (RDF or g(r)) indicates that NNIP sufficiently reproduces the local structure predicted by the AIMD simulations and uncovers the oscillating structures at larger distances as well. Additionally, the RDF of the Na^+^–Cl^−^ pair in Panel (b) exhibits a first maximum position at 2.77 Å and a first minimum position at 4.27 Å, which are close to those reported recently^27,28^ using NNIP training employing a different protocol. This indicates that the presence of a cavity with a radius of 4.0 Å does not significantly affect the average liquid structure in the nearby bulk.

Just outside of the cavity, it is observed that there is a slightly higher density of the Cl^−^ ions than the Na^+^ cations. Integrating to a distance of 5.5 Å the coordination number of Cl^−^ is 0.7 higher than that of Na^+^. This leads to a dipole layer in the vicinity of a cavity of 4 Å size. When the solute ion is present at the center of the cavity as shown in Panel (c), however, both solvent ions exhibit charge-symmetrical behavior, as evidenced by the overlaps in the IS RDFs.

**Fig. 2:**
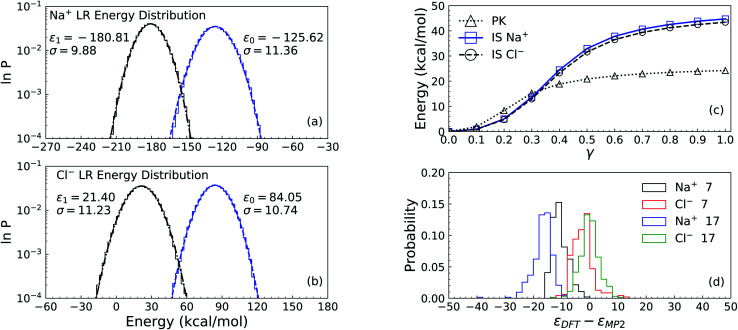
The process of excess chemical potential calculation for the systems of solute Na^+^ and Cl^−^ ions with 256 solvent ion pairs. Panel (a) and Panel (b) are for the long-ranged (LR) contributions to the solvation free energy of the solute ions Na^+^ and Cl^−^, respectively. The logarithms of the distribution of interaction energies from uncoupled (right, blue) and coupled (left, black) configurations are presented with the mean value *ε* and standard deviation *σ*. The right dashed curves and the left dash-dotted curves are the Gaussian fit to both distributions. Panel (c) is for the packing (PK) and minus the Inner-Shell (IS) cumulative contributions for both solute ions. Along with the expansion of the cavity with(IS)/without(PK) the solute ions at the center, the coupling parameter *γ* increases from 0 to 1. The IS and PK contributions are listed in Table 1. Panel (d) is the distributions of interaction energy corrections *ε*_DFT_ − *ε*_MP_2__ for the solute ion with 7 and 17 solvent ion pairs. The sampling is over 400 cluster configurations carved from DFT simulation trajectories. The DFT calculation is under Periodic Boundary Conditions (PBC) in a cell of size 25.4 Å.

The LR contribution is estimated from the two distributions of solute–solvent ion interaction energies, which are calculated over configurations from uncoupled and coupled simulations. As shown in Panel (a) of Fig. 2 for the solute Na^+^, the mean uncoupled interaction energy is −125.62 kcal mol^−1^ with a fluctuation contribution (eqn (9)) of 28.2 kcal mol^−1^, while the mean coupled interaction energy is −180.81 kcal mol^−1^ with a fluctuation contribution of 21.3 kcal mol^−1^. The difference between the two fluctuation terms leads to an error of 3.5 kcal mol^−1^ for the LR free energy contribution, indicating that the Gaussian approximation is inappropriate and a larger cavity is required to observe Gaussian behaviour as was previously seen in the calculation of the hydration free energy of the Na^+^ ion in water.^44,45^

As discussed in the theoretical methods section above, however, the long-range contribution is equal to the interaction energy at the intersection point of the two distributions. Thus we estimate the long-ranged contribution for Na^+^ as −156.0 kcal mol^−1^. In Panel (b) of Fig. 2, the distributions for the Cl^−^ ion exhibit more closely Gaussian behavior since the fluctuation terms are closer: 25.2 kcal mol^−1^ for uncoupled simulation with a mean value of 84.05 kcal mol^−1^ and 27.6 kcal mol^−1^ for coupled simulation with mean value of 21.40 kcal mol^−1^.

Based on the estimation of the intersection point of the two energy distributions, we estimate the LR contribution for Cl^−^ ion as 54.0 kcal mol^−1^. The dramatic increase of the LR contribution (including the sign) for the anion is attributed to the electrostatic potential at the center of the cavity.^45^ Assuming that the electrostatic interaction dominates the interactions between the center solute ion and solvent ions outside the cavity of 4 Å, the average energies of both Na^+^ and Cl^−^ ions over the uncoupled configurations gives an estimate of the electrostatic potential at the center of the cavity as −4.55 Volt, relative to the average potential in the bulk region of molten salt liquids.

Multipole electrostatic moment analysis of the cavity-water interfacial potential^69^ reveals that there is a potential shift of −3.96 Volt from the liquid water bulk phase to the cavity center, which is primarily attributed to the water molecular Bethe potential (quadrupole) contribution. Since the current work focuses on the excess free energy for the solute ion pair (in which case the interfacial potential contributions cancel exactly), the Bethe potential for liquid NaCl is not discussed further here.

The numerical results for the IS and PK contributions are shown in Panel (c) of Fig. 2 by presenting the cumulative work computed during the NNIP-MD simulations. Both Na^+^ and Cl^−^ ions share the same PK contribution (24.2 kcal mol^−1^), while the IS contribution to the Na^+^ solvation free energy is −44.7 kcal mol^−1^ and that for the Cl^−^ ion is −43.6 kcal mol^−1^.

The numerical results for both the Na^+^ and Cl^−^ ions are listed in Table 1. The finite-size correction term is due to the artificial effect of the Ewald potential on the free energy. It is shown to be 
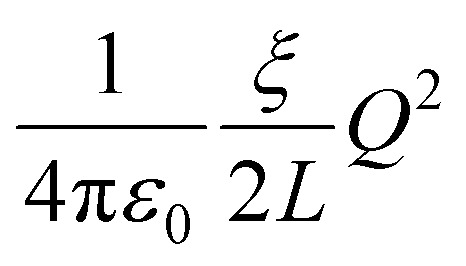
, where *Q* is the net charge in unit of elementary charge *e*, *L* is the box size, *ξ* = −2.837 297 for a cubic lattice and 
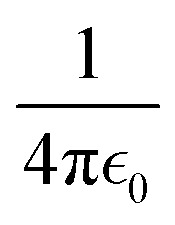
 = 332.063301 kcal mol^−1^ Å^−1^ × 10^2^.^43^ The summation of the excess free energy for both solute ions with 256 solvent ion pairs is −178.5 kcal mol^−1^, which is converged to within the standard error of the mean (SEM) of ±1.1 kcal mol^−1^ as the system size increases to 512 ion pairs. The SEM in parentheses is calculated using the block-average method (10 blocks) over the production configurations. To the best of our knowledge, this is the first prediction of the absolute solvation free energy for ions in a molten salt using deep learning techniques.

**Table 1:** The excess chemical potentials (kcal mol^−1^) for the Na^+^ ion and the Cl^−^ ion in molten NaCl at 1150 K. The second column is the packing contribution. The third column is the inner-shell contribution. The fourth column is the long-ranged contribution, and the fifth column is the finite-size correction (FS). The sixth column is the excess chemical potential of each solute ion and solute ion pair. The number of solvent ion pairs is given after “NaCl”. In the parentheses is the standard error of the mean (SEM) over 10 blocks. The last two columns give an estimate of the computational cost (core-hour/atom/MD-step) for the PK contribution where *γ* = 0.1. The calculations were performed on the OSC Pitzer cluster (Dual Intel Xeon 6148s Skylakes 2.4 GHz and 192 GB RAM). AIMD used 400 cores (4 nodes) and NNIP-MD used 40 cores (1 node)

Solute	*μ* ^PK^_X_	*μ* ^IS^_X_	*μ* ^L*R*^_X_	FS	*μ* ^ex^_X_	AIMD	NNIP-MD
Na^+^	25.4(0.2)	−44.8(0.2)	−142.8(0.7)	−28.8	−191.0(0.8)		
Cl^−^	25.4(0.2)	−43.8(0.2)	63.6(0.7)	−28.8	16.4(0.7)		
NaCl-64					−174.6(1.1)	1.5 × 10^−3^	5.5 × 10^−7^
Na^+^	24.5(0.3)	−44.6(0.2)	−149.2(0.7)	−23.1	−192.4(0.8)		
Cl^−^	24.5(0.3)	−43.7(0.2)	58.0(0.7)	−23.1	15.7(0.8)		
NaCl-128					−176.7(1.1)	3.6 × 10^−3^	2.9 × 10^−7^
Na^+^	24.2(0.2)	−44.7(0.2)	−156.0(0.7)	−18.4	−194.9(0.8)		
Cl^−^	24.2(0.2)	−43.4(0.2)	54.0(0.7)	−18.4	16.4(0.8)		
NaCl-256					−178.5(1.1)	4.0 × 10^−3^	2.7 × 10^−7^
Na^+^	24.3(0.2)	−44.4(0.2)	−158.8(0.7)	−14.7	−193.6(0.8)		
Cl^−^	24.3(0.2)	−43.6(0.2)	48.4(0.7)	−14.7	14.4(0.8)		
NaCl-512					−179.1(1.1)	6.6 × 10^−3^	2.5 × 10^−7^

The computational cost by AIMD and NNIP-MD are shown in the last columns of Table 1, where it is shown that the efficiency of the NNIP-MD simulation is about 3 orders greater than that of AIMD for all the molten salt systems and the SEMs are reduced by about 1 order of magnitude compared to the calculation reported.^42^ The AIMD cost for 128, 256 and 512 systems are estimated over 100 MD-steps.

**Fig. 3:**
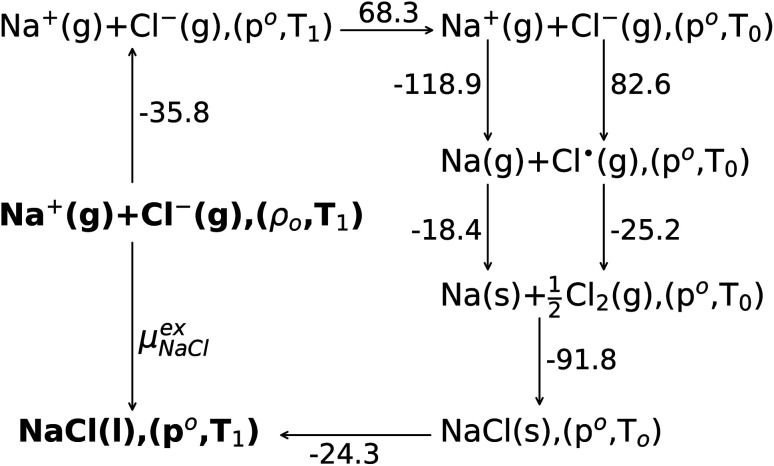
The Born–Haber cycle scheme for Na^+^ and Cl^−^ ion solvation. The ions are assumed to be solvated from the ideal gas state (g) to liquid state (l) with number density *ρ*_*o*_ = 15.61(nm)^−3^ and *T*_1_ = 1150 K (NVT ensemble). The ideal gas of ions are changed into the NPT ensemble with pressure *ρ*_o_ = 1 bar and temperature of 1150 K. Then the temperature of the ideal gases is reduced to *T*_0_ = 298.15 K isobarically. At room temperature the sodium ion is changed to the sodium atom and chloride ion to the chlorine radical. Both atoms are then changed into elements, respectively. The solid state (s) NaCl is formed from component elements at room temperature and 1 bar pressure. Then the solid salt are heated isobarically into the liquid phase (l) at *T*_1_ = 1150 K. The change of free energy from the NVT ensemble (*ρ*_o_,*T*_1_) to the NPT ensemble (p_o_,*T*_1_) of the liquid molten NaCl(l) is neglected.^43^ All of the Gibbs free energy changes are from thermodynamic tables,^67,68^ and the total change of the solvation free energy is −163.5 kcal mol^−1^.

The experimental data are analyzed in terms of a Born–Haber cycle as illustrated in Fig. 3. Assuming that all the gas states are well-approximated by the ideal gas, the sum of the solvation free energies of both solute ions is calculated from thermochemical tables^67,68^ to be −163.5 kcal mol^−1^. Consequently our QCT calculation at the DFT level over-estimates the excess Gibbs binding free energy by roughly −15 kcal mol^−1^.

In a previous study by Gray-Weale *et al.*,^42^ it was noted that the DFT calculation over-estimates the Na atom solvation free energy in liquid Na by −19 kcal mol^−1^ and the total Gibbs free energy change for the redox reaction Na(l)+ 
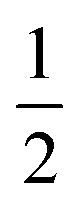
 Cl_2_(g) ⇌ NaCl(l) by −15 kcal mol^−1^. The molten salt density is over-estimated by up to 10% when using the PBE DFT functional with the D2 dispersion correction, while the system appears to be unstable without the D2 correction. These results indicate both the importance and the subtlety of dispersion forces in the molten salt liquids. It is not surprising that the over-estimation of the density correlates with the over-estimation of the magnitude of the excess free energy. Some over-binding is also observed in ion solvation for the aqueous solution.^45^

Herein to estimate the correction for the free energy based on the underlying DFT simulations, we implement higher-level density-fitted MP2 theory^70^ calculations over 400 cluster configurations with the basis set aug-cc-pvdz^71,72^ in the Psi4 package.^73^ The cluster configurations with 7 and 17 solvent ion pairs are carved from a trajectory generated by the DFT simulation.

The correction (in the direction DFT → MP2) to the solvation free energy of each ion Δ*μ*^ex^ is given by13
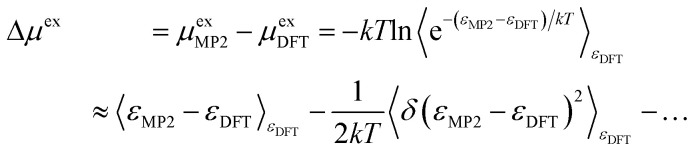
where the first term is the interaction energy correction and the second term is the fluctuation correction. The subscript *ε*_DFT_ indicates that the sampling is over configurations produced by the DFT simulation. As presented in Table 2, the results of DFT calculations (with the same basis set, pseudo-potential and functional as those in the above simulation) are close to the results of the MP2 calculations obtained with the Psi4 code for the sampled clusters. (Note that in Table 2 the results are presented in reference to the MP2 data; thus, the sign of the energetic correction should be flipped when inserting the data into eqn (13).)

Inclusion of the D2 correction over-estimates the interaction energy by −6.4 kcal mol^−1^ on average. As mentioned above, the over-binding between ions is also indicated by the 7% over-estimation of the molten NaCl density at 1150 K.^42^ The PBC interactions (related to the Ewald potential^45,62,63^) for the simulation cell of size 16.0 Å contributes about 10.0 kcal mol^−1^ to the interaction energy correction for the solute ion pair, while in the cell of size 25.4 Å it contributes about 2.0 kcal mol^−1^. The distributions of the interaction energy corrections under PBC in the cell of size 25.4 Å are presented in Panel (d) of Fig. 2.

To estimate the total free energy correction, including contributions from outside the clusters, we extrapolate the cluster interaction energy correction to 256 ion pairs. Considering the cancellation of Ewald potential contributions for the solute ion pair, we assume that it is convergent to 1.6 (1.4) kcal mol^−1^. Assuming the dispersion interaction energy 
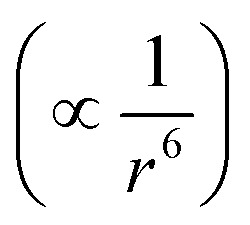
 is proportional to the solvent ion density (which is approximately a constant outside the first solvation shell), the spherical integration leads to the expression of dispersion energy correction as *a* + *b*/*N*, where N is the solvent ion pair number. Using the dispersion energy corrections presented in row number 2 of Table 2, we calculate the parameters *a* and *b* as (−11.59, 20.23) for Na^+^ and (−8.88, 16.66) for Cl^−^, which leads to the extrapolation for the dispersion energy correction as −11.5(1.1) kcal mol^−1^ for Na^+^ and −8.8(0.8) kcal mol^−1^ for Cl^−^ (in the MP2 → DFT direction). The total dispersion energy correction for the solute ion pair is then −20.3(1.4) kcal mol^−1^ or +20.3 kcal mol^−1^ in the desired DFT → MP2 direction. The Ewald correction in this direction is −1.6 kcal mol^−1^.

**Table 2:** Corrections for the interaction energy of the solute ion (Na^+^ or Cl^−^) with solvent ion pairs (7 and 17). The correction is referenced to the MP2 calculation using the Psi4 quantum chemistry package. In parenthesis is the SEM. In the square bracket is the standard deviation *σ* of the sampling. The configurations are carved out from a CP2K simulation trajectory without a cavity around the solute ion. The DFT calculations are implemented *via* the CP2K package. The MP2 and the first two DFT calculations are for the isolated systems. The subsequent two DFT calculations are under Periodic Boundary Condition (PBC) in the simulation cell of size 16.0 Å and 25.4 Å, respectively. The row number 5 shows the dispersion correction of D2 contributions. The PBC effects on the corrections are presented in the last two rows

	Na^+^ 07	Cl^−^ 07	Na^+^ 17	Cl^−^ 17
0) MP2	0.0	0.0	0.0	0.0
1) DFT	−1.9(0.5)[2.3]	−0.7(0.4)[3.5]	−3.1(0.4)[4.7]	−1.9(0.4)[3.9]
2) DFT + D2	−8.7(0.6)[2.8]	−6.5(0.5)[3.5]	−10.4(0.5)[5.3]	−7.9(0.3)[4.8]
3) DFT + D2 PBC 16.0	−15.9(0.6)[4.2]	10.8(0.5)[4.4]	−31.3(0.6)[9.6]	23.6(0.7)[7.0]
4) DFT + D2 PBC 25.4	−10.5(0.6)[2.8]	−2.5(0.4)[3.5]	−16.7(0.5)[3.4]	0.0(0.4)[3.4]
5) D2, (2)-(1)	−6.8(0.8)	−5.8(0.6)	−7.3(0.6)	−6.0(0.5)
6) PBC 16.0,(3)-(2)	−7.2(0.8)	17.3(0.7)	−20.9(0.8)	31.5(0.8)
7) PBC 25.4,(4)-(2)	−1.8(0.8)	4.0(0.6)	−6.3(0.7)	7.9(0.5)

Summing up the total correction (PBC/Ewald and dispersion) of the solute ions, we see that the correction from the cluster calculations with 17 solvent ion pairs dominates 89.3% of that with 256 solvent ion pairs. As a result, the fluctuation term in eqn (13) is assumed to be primarily captured by the interaction energy correction of 17 solvent ion pair cluster. This leads to a −2.5(0.1) kcal mol^−1^ correction for each of the Na^+^ and Cl^−^ solute ions. Inserting these results into eqn (13), we obtain the total correction (in the DFT → MP2 direction) as +13.7(1.9) kcal mol^−1^ for the solute ions. Compared to the experimental reference value of −163.5 kcal mol^−1^, the calculated total excess chemical potential of −164.8(2.2) kcal mol^−1^ provides strong initial validation of the methodology.

## 4 Conclusions

Through investigation of the solvation thermodynamics of the Na^+^ and Cl^−^ ions in the molten NaCl liquid, we have presented and validated an efficient and general methodology to calculate the solvation free energy of ionic species in the molten salts. The methodology incorporates *ab initio* molecular dynamics simulations, interatomic potentials based on deep neural network models, and quasichemical theory. Efficient molecular dynamics simulations with the NNIP model reduces the calculation uncertainty significantly to roughly 1 kcal mol^−1^. Due to the over-estimation of the magnitude of the attractive interaction energy of the solute ion with the solvent ions at the DFT level (with D2 dispersion corrections), a high-level quantum chemical correction of roughly 15 kcal mol^−1^ to the free energy is required to make a quantifiable prediction of the excess free energy. These results highlight the importance of dispersion and polarization interactions in the molten salt liquids.

The methodology sets the stage for larger-scale simulations of molten salt mixtures at a quantum mechanical level of accuracy, allowing for quantitative investigations of the activities, solubilities, and redox potentials of ionic species (including the corrosion and fission products) in the liquid phase.^40–42^ The methodology holds the potential to provide essential data for molten salt applications in both concentrated solar energy storage materials and molten salt reactors.

## Data availability

Data related to the calculations presented in this paper will be made freely available upon request to the authors.

## Author contributions

Yu Shi: conceptualization, methodology, software, validation, investigation, data curation, visualization, writing – original draft. Stephen T. Lam: methodology, validation, writing – review&editing. Thomas L. Beck: conceptualization, methodology, supervision, writing – review&editing, project administration, funding acquisition.

## Conflicts of interest

There are no conflicts to declare.

## Notes

This manuscript has been authored in part by UT-Battelle, LLC, under contract DE-AC05-00OR22725 with the US Department of Energy (DOE). The publisher acknowledges the US government license to provide public access under the DOE Public Access Plan (https://energy.gov/downloads/doe-public-access-plan).

## Supplementary Material
